# Cranial Growth and Variation in Edmontosaurs (Dinosauria: Hadrosauridae): Implications for Latest Cretaceous Megaherbivore Diversity in North America

**DOI:** 10.1371/journal.pone.0025186

**Published:** 2011-09-28

**Authors:** Nicolás E. Campione, David C. Evans

**Affiliations:** 1 Department of Ecology and Evolutionary Biology, University of Toronto, Toronto, Ontario, Canada; 2 Department of Palaeobiology, Royal Ontario Museum, Toronto, Ontario, Canada; Raymond M. Alf Museum of Paleontology, United States of America

## Abstract

The well-sampled Late Cretaceous fossil record of North America remains the only high-resolution dataset for evaluating patterns of dinosaur diversity leading up to the terminal Cretaceous extinction event. Hadrosaurine hadrosaurids (Dinosauria: Ornithopoda) closely related to *Edmontosaurus* are among the most common megaherbivores in latest Campanian and Maastrichtian deposits of western North America. However, interpretations of edmontosaur species richness and biostratigraphy have been in constant flux for almost three decades, although the clade is generally thought to have undergone a radiation in the late Maastrichtian. We address the issue of edmontosaur diversity for the first time using rigorous morphometric analyses of virtually all known complete edmontosaur skulls. Results suggest only two valid species, *Edmontosaurus regalis* from the late Campanian, and *E. annectens* from the late Maastrichtian, with previously named taxa, including the controversial *Anatotitan copei*, erected on hypothesized transitional morphologies associated with ontogenetic size increase and allometric growth. A revision of North American hadrosaurid taxa suggests a decrease in both hadrosaurid diversity and disparity from the early to late Maastrichtian, a pattern likely also present in ceratopsid dinosaurs. A decline in the disparity of dominant megaherbivores in the latest Maastrichtian interval supports the hypothesis that dinosaur diversity decreased immediately preceding the end Cretaceous extinction event.

## Introduction

The pattern of dinosaur diversity leading to the terminal Cretaceous extinction event continues to be hotly debated, with the well-sampled fossil record of North America forming the basis for differing hypotheses [1]–[6]. A core issue of this debate focuses on whether the diversity of dinosaurs either decreased from the Campanian through to the Maastrichtian [4]–[8] or remained relatively stable [1]–[3]. Due to the relatively low standing diversity of dinosaurs at any given time and geographic region, alpha level taxonomy plays a particularly important role in assessing patterns of diversity through this interval. Recent studies have underscored the importance of understanding ontogenetic and individual variation when considering the nature of dinosaur diversity, and have suggested that some dinosaur groups were less diverse in the late Maastrichtian than previously thought [9]–[12].

**Figure 1:**
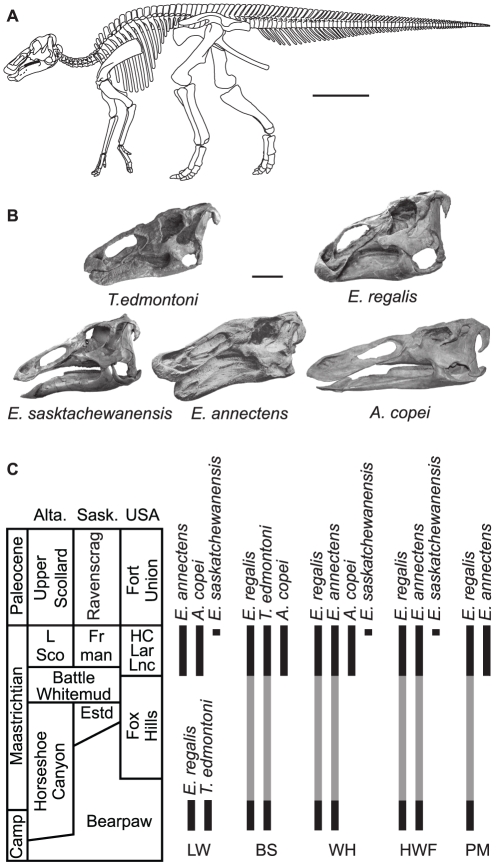
Type skulls and current biostratigraphic distributions of North American edmontosaurs. (A) Skeleton reconstruction of *Edmontosaurus regalis*. Scale bar, 100 cm. (B) Type skulls of the five named edmontosaur taxa: *Thespesius edmontoni* (CMN 8399), *Edmontosaurus regalis* (CMN 2288), *E. saskatchewanensis* (CMN 8509), *E. annectens* (YPM 2182, from [15]), *Anatotitan copei* (AMNH 5730). Scale bar, 20 cm. (C) Biostratigraphic distributions of edmontosaur species (in black) based on published synonymies. Grey bars indicate ghost ranges inferred by proposed taxonomic schemes. Abbreviations: Alta, Alberta; BS, Brett-Surman [35]; Camp, Campanian; Estd, Eastend Formation; Fr man, Frenchman Formation; HC, Hell Creek Formation; HWF, Horner et al. [13]; L Sco, Lower Scollard Formation; Lar, Laramie Formation; Lnc, Lance Formation; LW, Lull and Wright [15]; PM, Prieto-Márquez [36]; Sask, Saskatchewan; WH, Weishampel and Horner [14].

Hadrosaurine hadrosaurids (Ornithopoda) closely related to *Edmontosaurus* are among the most common dinosaurs in the late Campanian and Maastrichtian deposits of western North American, and are one of the few groups of large-bodied dinosaurs (body mass >1000 kg) currently thought to have undergone a radiation in the Maastrichtian, just prior to the end of the Cretaceous [13], [14]. Based on numerous complete specimens, five taxa have been historically recognized (Figure 1) [15]: two based on type material from the upper Campanian of Alberta, Canada (Horseshoe Canyon Formation), *Edmontosaurus regalis* and *Thespesius edmontoni*; and three based on type material from the upper Maastrichtian of the western interior (Hell Creek, Lance, and equivalent formations), *E. saskatchewanensis*, *E. annectens*, and *Anatotitan copei*. In general, *E. regalis* and *E. annectens* are considered valid, but considerable debate regarding the validity of the other taxa has resulted in numerous opinions and synonymies (Figure 1 and Text S1) that have created considerable confusion in the biostratigraphic ranges of these species, with several schemes incurring species durations in excess of seven million years for both *E. annectens* and *E. regalis* (Figure 1C). Although the number of species recognized varies, all schemes infer an increase in diversity leading up to the end-Cretaceous extinction event. These species occurrences and synonymies are often proposed without reference to particular specimens and lack supporting character data to justify assignments. This confusing taxonomic history has led to uncertainty about the diversity of edmontosaurs, with as many as four morphologically, and presumably ecologically, similar species present in the late Maastrichtian [14].

**Figure 2:**
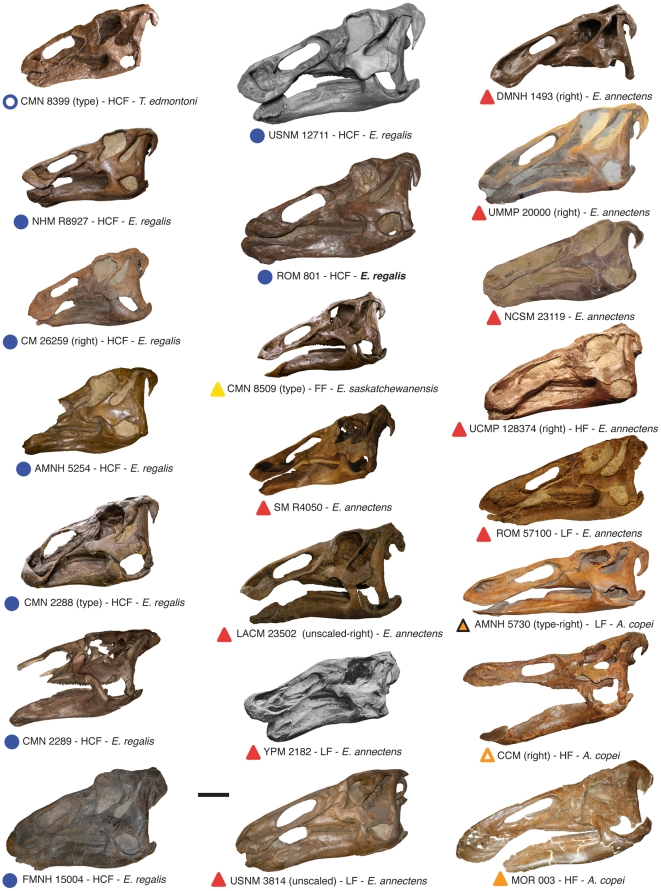
Compilation of virtually all known complete edmontosaur skulls from North America. All skulls are in lateral view (sometimes reversed). Labels below each skull include the symbol used in the morphometric plots, whether the specimen represents a holotype (type), the formation where it was uncovered (HCF, Horseshoe Canyon Formation; HF, Hell Creek Formation; FF, Frenchman Formation; LF, Lance Formation), and the species name based on traditional edmontosaur taxonomy [15]. Scale bar, 20 cm.

Published diagnoses emphasize subtle proportional differences in the skull as diagnostic features for edmontosaur species [15], [16], yet the potential influence of size, individual, and ontogenetic variation on these features has not been tested. In this paper, we address the issue of edmontosaur diversity by taking a rigorous morphometric approach to assess variation in the large sample of complete skulls (Figure 2). We revise the species-level diversity and biostratigraphic distribution of edmontosaurs, and discuss these results in the context of North American dinosaur diversity and disparity dynamics leading up to the end-Cretaceous extinction event.

## Materials and Methods

In order to understand the range of morphological variation in edmontosaur crania we compiled an extensive database of linear measurements and landmark data derived from the examination of virtually all known relatively complete skulls. The total dataset consists of 23 specimens (22 of them shown in Figure 2, as BHI 2169 is disarticulated; see Text S1 for institutional abbreviations). Of these, nine are from the late Campanian Horseshoe Canyon Formation, and the remaining specimens are from the latest Maastrichtian Hell Creek Formation or temporally equivalent strata. Skull length ranges from 781 mm, in *Edmontosaurus saskatchewanensis* (CMN 8509), to 1278 mm in *Anatotitan copei* (MOR 003, which is referred to this taxon based on its proportionately long, low skull morphology, [16]). Some taphonomic deformation of individual specimens is undoubtedly present in this dataset. However, to avoid the exclusion of particular data points on the basis of subjective assessments of taphonomic distortion, we have opted to include as many specimens as possible, and discuss potential preservation effects *a posteriori*.

**Figure 3:**
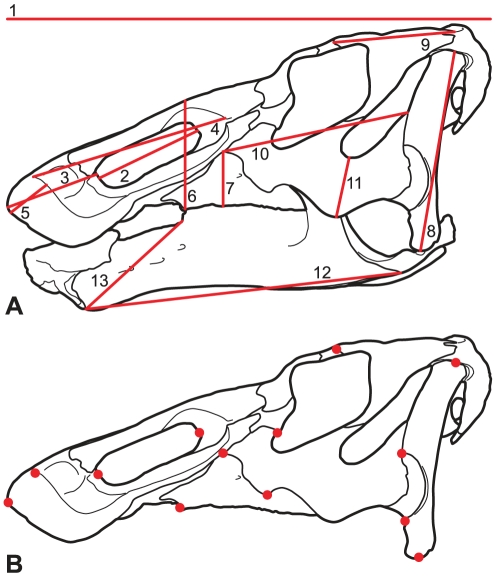
Measurements and landmarks used in this study. (A) The suite of 13 linear measurements taken and used in the principal component analysis and bivariate allometric analyses. Numbers correspond to those indicated in the table of measurements (Table S1). (B) The set of 13 landmarks used in the geometric morphometric analysis.

Thirteen linear measurements were chosen to describe each skull on the basis of their easy-to-constrain, repeatable nature, prevalence in the hadrosaurid literature [17], [18], putative diagnostic variability in edmontosaurs [15], and in order to minimize missing data in quantitative analyses (Figure 3A and Table S1). These measurements form a network that captures the overall shape of the skull while attempting to avoid unnecessary duplication that may overemphasize potential statistical and measurement error. Due to the high level of heteroscedasticity in the dataset, all variables were log-transformed. Variation in the linear measurement data was analyzed using a Principal Component Analysis (PCA). Some specimens are incomplete and therefore certain variables could not be measured. Missing values were estimated using the Bayesian Principal Component Analysis (BPCA) method [19]. Although missing data could be estimated for extremely poor specimens, we include only those for which at least 50% of the measurements could be confidently obtained (N = 21). Linear measurements were also used in a series of bivariate plots and reduced major axis (RMA) analyses to describe relative growth in the skulls of edmontosaurs as they relate to skull length. RMA lines were calculated for the entire dataset as well as for the late Campanian and late Maastrichtian subsamples separately.

This study also employs a geometric morphometric (GM) approach based on 13 landmarks obtained from lateral views of the skulls (Figure 3B). Only complete skulls could be analysed using GM (N = 17). The landmarks were placed directly on the photographs using the software TpsDig2 [20]. The majority of photographs were taken using a Canon Rebel XS and an 18–55 mm lens (by NEC); however, some specimens were photographed by other individuals. TpsRelw [21] was used to determine the Relative Warp Scores. GM plots were created using the statistical programming language R [22], except for the vector plots, which were created with the program PCAGen6, in the IMP package [23].

Box and whisker plots were created to visually inspect for statistical outliers and to visualize the range of temporally equivalent specimens along the taxonomically relevant principal component/relative warp axes, as determined by the multivariate analyses. In both the linear PCA and GM, the Campanian and Maastrichtian subsamples are normally distributed (Shapiro-Wilks Test: p>0.05), and therefore statistical differences between temporal clusters were tested using a two-tailed t-test. All multivariate and bivariate analyses of linear measurements were performed in R [22], with the packages MASS [24], lmodel2 [25], and pcaMethods [26].

In order to address patterns of hadrosaurid diversity and disparity leading up to the end of the Cretaceous, we compiled a database of complete hadrosaurid crania known from North America, spanning the latest Campanian to the end of the Maastrichtian (73 to 65.5 Ma). Three equal time bins were used to account for the uncertainty in species occurrences (e.g., Lance Formation), and the duration of the bins was selected to confidently accommodate the entire duration of the Lancian time interval (approx. 68 to 65.5 Ma; [27]), the highest temporal resolution possible given the given the available biostratigraphic data.

Because the number of species in each time bin depends on alpha taxonomy, which is often based on contentious interpretations, we quantify changes in morphological disparity using Foote's disparity metric calculated for hadrosaurid assemblages as a proxy for ecological diversity through the three time intervals [28]–[30]. Disparity was estimated based on 12 landmarks including all complete hadrosaurid skulls from the three selected time intervals (Figure S3). Morphological disparity (MD) was calculated using the IMP7 software package and the module DisparityBox7 [23]. Two issues needed to be addressed in order to get a complete picture of hadrosaurid MD during the latest Cretaceous. 1) Only a single subadult lambeosaurine skull is known from the latest Campanian interval, *Velafrons coahuilensis* from the Cerro del Pueblo Formation [31]. This specimen has been interpreted as immature and, therefore, based on previous observations that the morphology of the crest is positively allometric [18], it is likely that the crest of the holotype of *V. coahuilensis* does not reflect the full adult morphology. In order to better approximate the disparity during the latest Campanian interval, in addition to the holotype of *V. coahuilensis*, we have included an adult *Hypacrosaurus altispinus* (CMN 8501), a close relative of *V. coahuilensis*, as a close approximation to the adult form. 2) During the early Maastrichtian interval *H. altispinus* and *Saurolophus osborni* are the only hadrosaurids known from complete cranial material. However, based on ghost ranges, as well as the juvenile specimens discovered in the Prince Creek Formation of Alaska [32], an *Edmontosaurus* species must be present during this time. Because the actual affinities of Alaskan material remain to be determined we calculated the disparity of the early Maastrichtian with the use of *E. regalis* specimens (MD = 0.0614) and then with specimens of *E. annectens* (MD = 0.0526). Both provide similar disparity estimates and as a result we have opted to use the results from the analysis with *E. regalis*, as this taxon, although not temporally equivalent, is known from the same formation (Horseshoe Canyon) as *H. altispinus* and *S. osborni*.

## Results

**Figure 4:**
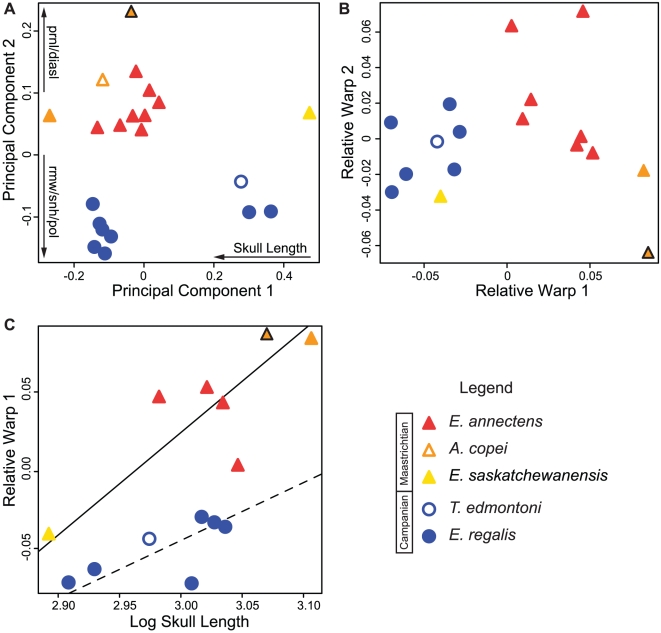
Multivariate and geometric morphometric results. (A) Plot of the first and second principal component axes (87.3% of the total variation) from the PCA of linear measurements. Arrows show the direction of increase along a PC axis of particular variables. (B) Plot of the first and second relative warps from the geometric morphometric analysis (65% of the total variation). (C) RMA analysis of relative warp 1 (RW1) against skull size. Solid and dashed lines represent the Maastrichtian and Campanian subsamples, respectively (Table 1). The orange triangle with black outline represents the holotype of *A. copei* (AMNH 5730), and the orange triangle represents the largest skull in the dataset (MOR 003), here assigned to *A. copei*. Abbreviations: diasl, length of edentulous portion of the dentary; pol, postorbital length; prnl, prenarial length; rmw, width of reflected margin of premaxilla; snh, snout height.

In the linear PCA, PC1 (65.9%) and PC2 (21.4%) represent 87.3% of the total variation in the variables. All the variables increase towards the negative end of PC1 (Table S2), and indicate that skull size is a major influence on the variation in this axis. Variation along the second principal component axis is largely independent of size. The remaining 11 axes each represent <5% of the overall variation and were not considered further. Variation along the second principal component, from the negative to the positive spectrum, is associated with a lengthening of the rostral region of the skull (prenarial length, diastema length, and narial vestibule length), and a shortening of the reflected margin of premaxilla, the height of snout, the length of postorbital, and the height of maxilla (Table S2). The PCA plot (Figure 4A) reveals that all specimens associated with the positive side of the second axis are from late Maastrichtian time, whereas all of the specimens from the latest Campanian Horseshoe Canyon Formation are negatively associated with the second axis. Based on the distribution of specimens along PC2, the Campanian and Maastrichtian temporal subsamples are significantly different from each other (t = −9.541, p≪0.01).

The first two relative warp axes of the geometric morphometric analysis represent 65% of the total variation (Figure 4B). The variation related to the first relative warp (46.5% of the variation), from the negative spectrum to the positive, is associated with an increase in snout length and an overall decrease in relative skull height (Figure S1A). Late Maastrichtian and latest Campanian samples segregate along RW1, with the latter occupying the negative end of the spectrum. The only exception to this pattern is the position of the smallest skull in the dataset, *E. saskatchewanensis* (CMN 8509), which plots near the late Campanian sample (Figure 4B). Despite this, the distributions of the temporal subsamples remain significantly different along RW1 (t = 5.9261, p≪0.01). The variation in RW2 (18.8% of the total variance) is largely related to rotation of the temporal region of the skull and is associated with the position of the quadrate (Figure S1B); there is no taxonomic or temporal clustering along this axis. The quadrate in hadrosaurids is loosely integrated into the skull and is mobile [33], making RW2 difficult to interpret due to the possible influence of taphonomic factors.

**Figure 5:**
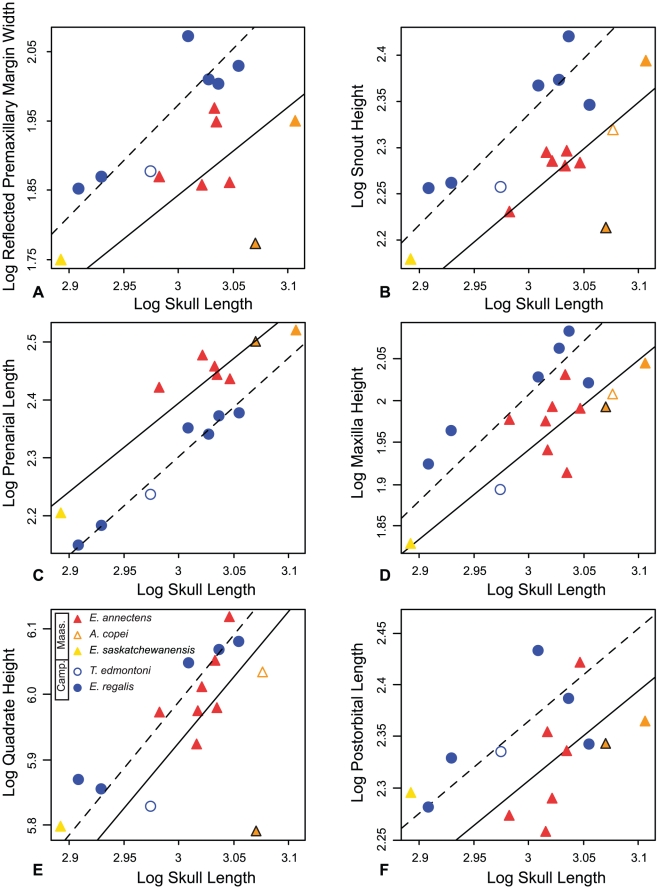
Bivariate allometric results. Bivariate plots and RMA lines for a variety of skull measurements against skull length (proxy for size) comparing late Campanian (circles/dashed lines) and late Maastrichtian (triangles/solid lines). (A) Width of the reflected margin of the premaxilla, (B) snout height, (C) length of prenarial region, (D) maxilla height, (E) Quadrate height, and (F) postorbital length. The orange triangle with black outline represents the holotype of *A. copei* (AMNH 5730), and the orange triangle represents the largest skull in the dataset (MOR 003). RMA statistics are presented in table 1.

Reduced major axis lines reveal that all variables are correlated with skull size (Figure 5 and Table 1). When the Maastrichtian and Campanian samples are analyzed separately, most plots show some segregation of the two subsamples, and in general, these RMA lines have higher coefficients of determination than that of the pooled sample. The late Campanian and late Maastrichtian samples exhibit positive allometry of the prenarial region of the snout. However, for a given size, it appears that the late Maastrichtian sample exhibits a proportionally longer prenarial region than in the late Campanian specimens. Most variables, such as dorsoventral snout height and reflected margin of the premaxilla, are statistically isometric in both late Maastrichtian and late Campanian specimens but show that for a given size there are notable differences between the subsamples. In these latter two plots, as well as many others, late Campanian and late Maastrichtian trends diverge with increasing skull size (Figure 5).

**Table 1:** Results from the bivariate allometric analyses.

Variable (*y*)	Sample	N	Slope (*m*)	95% CI *m*	Intercept (*b*)	95% CI *b*	R^2^	Trend
Relative Warp 1	All	14	0.915	0.584 to 1.432	−2.753	−4.307 to −1.761	0.457	-
	Campanian	7	0.364^ns^	0.171 to 0.773	−1.134	−2.355 to −0.559	0.482	-
	Maastrichtian	7	0.644	0.346 to 1.197	−1.906	−3.58 to −1.007	0.669	-
Reflected Margin of Premaxilla	All	15	1.541^ns^	0.922 to 2.577	−2.724	−5.838 to −0.86	0.19	iso
	Campanian	7	1.606	0.93 to 2.772	−2.844	−6.331 to −0.823	0.751	iso
	Maastrichtian	8	1.258^ns^	0.585 to 2.704	−1.929	−6.303 to 0.105	0.288	iso
Snout Height	All	17	1.114	0.719 to 1.726	−1.061	−2.905 to 0.129	0.325	iso
	Campanian	7	1.199	0.668 to 2.153	−1.261	−4.116 to 0.329	0.709	iso
	Maastrichtian	10	1.009	0.605 to 1.682	−0.779	−2.817 to 0.444	0.571	iso
Prenarial Length	All	15	1.964	1.497 to 2.576	−3.543	−5.384 to −2.139	0.79	pos
	Campanian	7	1.7	1.351 to 2.139	−2.797	−4.109 to −1.754	0.959	pos
	Maastrichtian	8	1.533	1.089 to 2.158	−2.204	−4.094 to −0.861	0.878	pos
Naris Length	All	14	1.221	0.885 to 1.686	−2.64	−2.64 to −0.232	0.728	iso
	Campanian	7	1.452	0.808 to 2.612	−1.926	−5.395 to 0.003	0.708	iso
	Maastrichtian	7	1.1	0.655 to 1.846	−0.885	−3.141 to 0.458	0.778	iso
Narial Vestibule Length	All	16	1.368	1.137 to 1.645	−1.441	−2.277 to −0.746	0.895	pos
	Campanian	7	1.386	0.955 to 2.01	−1.508	−3.375 to −0.22	0.89	iso
	Maastrichtian	9	1.197	0.942 to 1.521	−0.913	−1.894 to −0.141	0.927	iso
Maxilla Height	All	18	1.117	0.764 to 1.633	−1.385	−2.941 to −0.321	0.461	iso
	Campanian	7	1.274	0.642 to 2.529	−1.816	−5.569 to −0.075	0.585	iso
	Maastrichtian	11	1.071	0.708 to 1.621	−1.272	−2.935 to −0.173	0.681	iso
Quadrate Height	All	16	0.767	0.492 to 1.196	0.279	−1.01 to 1.106	0.359	iso
	Campanian	6	0.811	0.428 to 1.536	0.162	−2.003 to 1.305	0.758	iso
	Maastrichtian	10	0.8^ns^	0.406 to 1.575	0.17	−2.169 to 1.359	0.198	iso
Postorbital Length	All	15	0.868^ns^	0.52 to 1.449	−0.274	−2.022 to 0.772	0.202	iso
	Campanian	6	0.898^ns^	0.357 to 2.258	−0.328	−4.388 to 1.286	0.418	iso
	Maastrichtian	9	0.87^ns^	0.43 to 1.758	−0.303	−2.987 to 1.025	0.27	iso
Jugal Length	All	18	0.77	0.548 to 1.081	0.219	−0.718 to 0.887	0.574	iso
	Campanian	7	0.807^ns^	0.382 to 1.707	0.108	−2.583 to 1.381	0.49	iso
	Maastrichtian	11	0.749	0.464 to 1.208	0.283	−1.106 to 1.144	0.567	iso
Jugal Height	All	18	1.096	0.724 to 1.659	−1.273	−2.971 to −0.153	0.352	iso
	Campanian	7	1.107	0.686 to 1.786	−1.268	−3.298 to −0.009	0.813	iso
	Maastrichtian	11	1.147^ns^	0.643 to 2.048	−1.454	−4.179 to 0.073	0.341	iso
Dentary Length	All	15	1.02	0.755 to 1.379	−0.211	−1.292 to 0.588	0.74	iso
	Campanian	6	1.318^ns^	0.604 to 2.879	−1.103	−5.793 to 1.044	0.613	iso
	Maastrichtian	9	0.87	0.644 to 1.176	0.24	−0.685 to 0.924	0.883	iso
Diastema Length	All	15	1.279	1.003 to 1.631	−1.357	−2.413 to −0.528	0.833	pos
	Campanian	6	1.286	0.843 to 1.961	−1.392	−3.407 to −0.07	0.902	iso
	Maastrichtian	9	1.113	0.75 to 1.651	−0.844	−2.469 to 0.251	0.795	iso

RMA analyses of linear measurements against skull length (*x*). RMA formulas expressed as *logy = mlogx+b*. Positive or negative allometry is considered when the slope of the lines are significantly different from a slope of 1, as indicated by the 95% confidence intervals.

ns slope not significantly different from 0 (two-tailed t-test: p>0.05).

## Discussion

### Ontogeny and systematics of edmontosaurs

Results suggest consistent morphological differences between late Campanian and late Maastrichtian edmontosaur samples. Within these samples, proportional differences previously deemed diagnostic of distinct taxa are clearly correlated with skull size. This suggests that intraspecific allometry may have played an important historical role in the recognition of taxa and identification of individual specimens.

**Figure 6:**
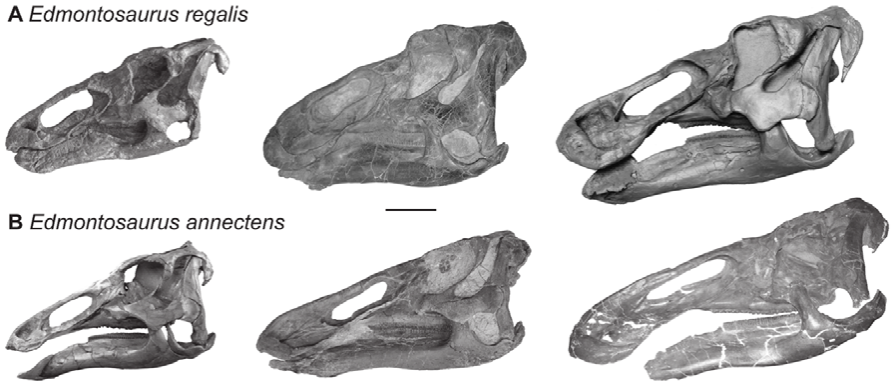
*Edmontosaurus* growth series. Hypothesized growth series for the two recognized *Edmontosaurus* species. (A) Specimens from left to right: CMN 8399 (holotype of *T. edmontoni*), USNM 12711, ROM 801. (B) Specimens from left to right CMN 8509 (holotype of *E. saskatchewanensis)*, ROM 57100, MOR 003. Scale bar, 20 cm.

Previous studies have identified two species, *Edmontosaurus regalis* and *Thespesius edmontoni*, from the late Campanian Horseshoe Canyon Formation of Alberta. *Thespesius edmontoni* is known from rare specimens and is generally smaller then the contemporaneous *E. regalis*. The holotype skull of *T. edmontoni* falls within the range of variation of *E. regalis* based on the multivariate results (Figure 4A,B); therefore *T. edmontoni* is interpreted here as a junior synonym of *E. regalis* [34], and not *E. annectens* as has often been suggested [13], [14], [35]. Morphological differences initially considered diagnostic for this taxon, including the height of the skull relative to its length and relatively small postorbital pocket (Figures 5 and 6A), show allometric variation consistent with the preserved size series of *E. regalis*, and similarities with *E. annectens* can be explained by the small size of *T. edmontoni* specimens.

Three species have been generally recognized from the late Maastrichtian: *Edmontosaurus annectens*, *E. saskatchewanensis*, and *Anatotitan copei*. The holotype and only exemplar of *E. saskatchewanensis* (CMN 8509) is the smallest, and presumably ontogenetically youngest, articulated individual in the database. In the linear PCA analysis this specimen groups with coeval late Maastrichtian specimens (PC 2), but in the GM analysis plots close to the *E. regalis* cluster (Figure 4A,B). Its small size suggests its placement in morphospace may be influenced by allometry, and potentially convergent morphology at small sizes (Figure 5). In order to assess potential allometric effects, RW1 was regressed against size. Because the GM results account for isometric scaling between specimens, any relationship of RW1 with size reflects, in part, allometric changes. When RW1 of late Maastrichtian and late Campanian specimens are regressed against skull length (Figure 4C and Table 1), the relationship is only significant in the late Maastrichtian sample (*m* = 0.644; R^2^ = 0.669; p<0.05). This indicates: 1) that the Maastrichtian sample is not isometric, and 2) that there is a greater similarity of form between temporal subsamples at small size. Here *E. saskatchewanensis* more closely resembles the pattern in the Maastrichtian sample, as in the linear PCA (Figure 4A). Bivariate plots also indicate that variables distinguishing late Maastrichtian and late Campanian samples (such as the size of the reflected margin of the premaxilla, the snout height, and the length of the postorbital) converge at small size (Figure 5). As a result, similarities of *E. saskatchewanensis* to *E. regalis* in the geometric morphometric analysis can be interpreted as resulting from its small size and probable subadult ontogenetic stage. Qualitative diagnostic characters further support the conclusion that CMN 8509 is a juvenile individual assignable to *E. annectens* [36], including a weakly developed excavation of the narial vestibule and postorbital ‘pocket’ (Figure 5).

The three longest skulls in the database are assigned to the taxonomically contentious *Anatotitan copei*. Chapman and Brett-Surman [16] diagnosed this taxon primarily on the basis of a long, low skull compared to other hadrosaurids. However, specimens of *A. copei* largely fall within (linear morphometrics), or very close to (geometric morphometrics), the range of variation in *E. annectens*, and its peculiar morphology can be adequately explained by allometric scaling of cranial proportions with size and individual variation within the late Maastrichtian sample (Figures 4C and 5). Although the holotype skull is unusually low in the linear analysis (PC 2), it is not an outlier in the GM analysis, and other specimens of *A. copei* fall within the expected range of the late Maastrichtian sample (Figures 4A,B and S2). Furthermore, as others have suggested, its unusual morphology is likely accentuated by dorsoventral crushing [13], [36].

Unfortunately it is impossible to test for fine-scale stratigraphic segregation of morphological variation within either of the latest Campanian or latest Maastrichtian intervals due to the absence of precise locality data for most edmontosaur specimens and the resulting lack of high-resolution biostratigraphic frameworks for these intervals. Regardless, the morphometric data presented here shows that any potential biostratigraphic trends within these two intervals would be subtle and difficult to distinguish from intraspecific allometric effects and individual variation within edmontosaur subsamples.

Morphometric analyses suggest strongly that cranial variation previously used to diagnose certain edmontosaur species can be explained by ontogenetic size increases and allometric growth within only two valid taxa: *Edmontosaurus regalis* from the latest Campanian (Figure 6A) of Alberta, and *E. annectens* from the late Maastrichtian of the western interior (Figure 6B). At large size (skull length >1 m), *E. regalis* is characterized by a rostrocaudally short and dorsoventrally tall snout, a well developed reflected margin of the premaxilla, a well excavated narial vestibule, and a deep postorbital ‘pocket’, relative to *E. annectens*. However, allometric trends of a number of diagnostic characters, including the thickness of the reflected margin of the premaxilla and the height of the snout, converge at small size, making the taxonomic assignment of small edmontosaur specimens problematic.

### Systematic Paleontology

Ornithischia Seeley 1887 [37]

Ornithopoda Marsh 1881 [38]

Hadrosauridae Cope 1869 [39]

Hadrosaurinae Cope 1869 [39]

*Edmontosaurus* Lambe 1917 [40]

#### Type Species

*Edmontosaurus regalis* Lambe 1917 [40]

#### Diagnosis

Hadrosaurine hadrosaurid characterized by the following autapomorphies: well-developed caudally directed reflected lateral margin of premaxilla; strongly excavated narial fossa along caudoventral margin of naris; well-developed fossa along orbital margin of prefrontal; large contribution of frontal to orbital margin; presence of a large fossa along orbital edge of the postorbital ( = postorbital ‘pocket’).

*Edmontosaurus regalis* Lambe 1917 [40]

*Thespesius edmontoni* Gilmore 1924 [41]

*Anatosaurus edmontoni* (Gilmore 1924) [41]: Lull and Wright [15] (new combination)

#### Holotype

CMN 2288, complete skull and partial postcranial skeleton; Red Deer River, Alberta, opposite the mouth of Three Hills Creek, 200 feet above the river level; Horseshoe Canyon Formation.

#### Paratype

CMN 2289, partial disarticulated skull and almost complete postcranial skeleton; west side of the Red Deer River, 7 miles northwest of Morrin, 90 feet above the river level; Horseshoe Canyon Formation.

#### Referred Specimens

All referred was collected along the Red Deer River, in the lower units of the Horseshoe Canyon Formation, Alberta. AMNH 5254, partial skull; NHM R8927, complete skull and postcranial skeleton; CM 26259, complete skull and partial postcranial skeleton; CMN 8399, complete skull and postcranial skeleton; CMN 8744, partial skull; FMNH 15004, complete skull; ROM 801, partial skull and postcranial skeleton; ROM 658, partial skull; ROM 867, partial skull and postcranial skeleton; USNM 127211, complete skull.

#### Diagnosis

Hadrosaurine hadrosaur, which at large size (skull length >1 m) is differentiated from *Edmontosaurus annectens* by the following characteristics: very wide, ‘swollen-like’ appearance to reflected margin of premaxilla; ventral expansion of rostral end of nasal; rostrocaudally short snout region rostral to naris; well-developed caudodorsal corner of narial fossa that extends above dorsal margin of skull; greater development of postorbital fossa; expansion of postorbital fossa results in horizontal shelf-like articular surface for postorbital on dorsal process of jugal.

#### Comments

Definitive occurrences of this species are restricted to latest Campanian strata of Alberta, Canada.

*Edmontosaurus annectens* (Marsh 1892) [42]

*Claosaurus annectens* Marsh 1892 [42]

*Anatosaurus annectens* (Marsh 1892) [42]: Lull & Wright [15] (new combination)

*Edmontosaurus annectens* (Marsh 1892) [42]: Horner et al. [13] (new combination)

*Thespesius saskatchewanensis* Sternberg 1926 [43]

*Anatosaurus saskatchewanensis* (Sternberg 1926) [43]: Lull and Wright [15] (new combination)

*Edmontosaurus saskatchewanensis* (Sternberg 1926) [43]: Horner et al. [13] (new combination)

*Diclonius mirabilis* Cope 1883 [44]

*Anatosaurus copei* Lull & Wright 1942 [15]

*Anatotitan copei* (Lull and Wright 1942) [15]: Chapman and Brett-Surman [16] (new combination)

#### Holotype

USNM 2414, partial skull roof and postcranial skeleton; north of Lightning and east of Bull creeks; Lance Formation.

#### Paratype

YPM 2182, south of Schneider and north of Greasewood creeks, near a smaller tributary of the Cheyenne River, Niobrara Co., Wyoming; Lance Formation.

#### Referred Specimens

AMNH 427, skull roof and braincase; AMNH 5046, partial juvenile skull, Sand Creek, Montana, Hell Creek Formation; AMNH 5060, complete skull and postcranial skeleton, Converse County, Wyoming, Lance Formation; AMNH 5046, partial juvenile skull (missing snout), Sand Creek, Montana, Hell Creek Formation; AMNH 5730, complete skull and postcranial skeleton, Moreau River, South Dakota, Lance Formation; BHI 2169, complete disarticulated skull; CCM No Catalogue Number, partial skull and complete postcranial skeleton, Montana, Hell Creek Formation; CMN 8509, complete skull and partial postcranial skeleton, Rocky Creek, Saskatchewan, Canada, Frenchman Formation; DMNH 1493, Dawson County, Montana, Hell Creek Formation; LACM 23502, complete skull, Garfield County, Montana, Hell Creek Formation; MOR 003, complete skull, Yellowstone County, Montana, Hell Creek Formation; MOR 1627, Glendive, Montana, Hell Creek Formation; NCSM 23119, complete skull, Carter County, Montana, Hell Creek Formation; ROM 57100, complete skull, Perkins County, South Dakota, Lance Formation; SM R4050, complete skull and postcranial skeleton; UCMP 128372, complete skull, Garfield County, Montana, Hell Creek Formation; UMMP 20000, complete skull, Garfield County, Montana, Hell Creek Formation; USNM 3814, complete skull and partial postcranial skeleton, Niobrara County, Wyoming, Lance Formation.

#### Diagnosis

Hadrosaurine hadrosaurid, which at large size (skull length >1 m) is differentiated from *Edmontosaurus regalis* by the following characteristics: presence of a very long prenarial region of skull; weakly excavated caudodorsal corner of narial vestibule; weakly developed postorbital fossa that results in a strait dorsal process of jugal.

#### Comments

Definitive occurrences of this species are restricted to latest Maastrichtian strata of western North America.

### Implications for edmontosaur biostratigraphy and evolution

**Figure 7:**
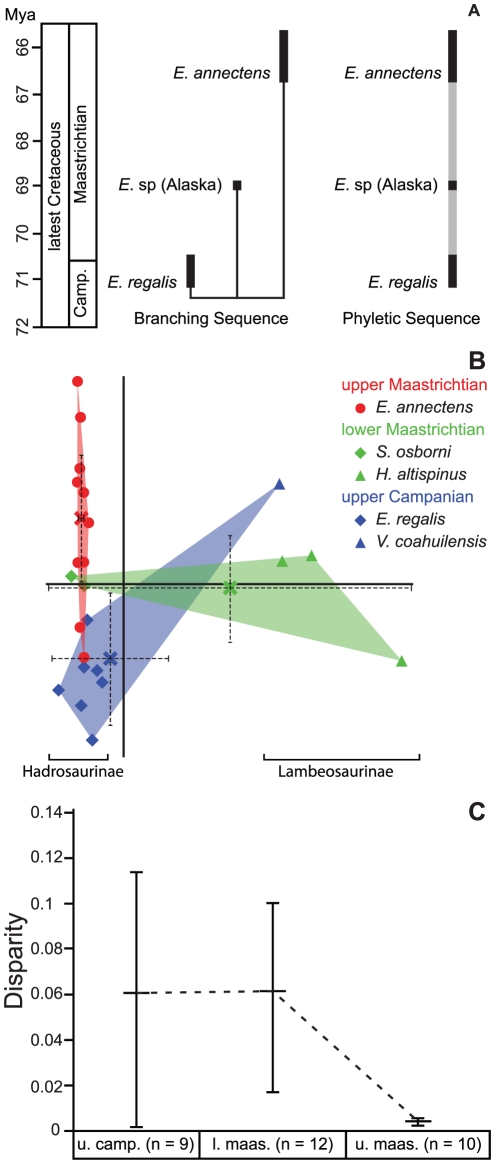
Biostratigraphy and evolution of edmontosaurs and hadrosaurid disparity during latest Cretaceous. (A) Revised biostratigraphic ranges of edmontosaur species during the latest Cretaceous. Based on our results either a cladogenetic (left) or anagenetic mode (right) of evolution is possible for this genus. (B) Results from the morphometric analysis including virtually all hadrosaurid skulls known from the latest Cretaceous (Figures 2 and S3A). The minimum convex polygons represent specimens known from the three time intervals described in the text. The centroid for each cluster and 95% confidence intervals is marked by an ‘X’ and the dotted lines. (C) Pattern of hadrosaurid morphological disparity, as measured by Foote's Disparity Metric, from 73 to 65 Ma, which shows a significant drop from the early to late Maastrichtian.

Published faunal lists have reported the presence of *Edmontosaurus regalis* in the upper Maastrichtian of Canada and the United States [13], [14], [36] and/or *E. annectens* in the uppermost Campanian [35]. However, our morphometric survey of virtually all relatively complete edmontosaur skulls finds no evidence of *E. regalis* in the Hell Creek or other generally coeval formations. Similarly, there is no unequivocal evidence of *E. annectens* in the late Campanian. A discriminant function analysis (of the linear measurements) based on an *a priori* designation of temporal samples corroborates a distinction between the late Campanian and late Maastrichtian samples (100% correct classification). Therefore, we eliminate the unusually long, seven million year biostratigraphic ranges for *E. regalis* and *E. annectens* reported in previous studies (Figures 1C and 7A).

Based on the non-overlapping biostratigraphic distribution and sister taxon relationship between *E. regalis* and *E. annectens* [36] we cannot reject the hypothesis that these two species have a phyletic relationship (e.g., [45]). Juvenile edmontosaur material from the Prince Creek Formation of Alaska [32] occur between the Horseshoe Canyon and late Maastrichtian edmontosaur samples (Figure 6A), as a result determining the affinities of these specimens may provide important insights into the pattern of edmontosaur evolution. All previous work on edmontosaur systematics has implied an overall increase in species richness from the late Campanian to the late Maastrichtian (Figure 1C). However, this study demonstrates at least a stable (taxic), or possibly decreasing (ghost ranges included), diversity dynamic in this clade of large-bodied primary consumers into the late Maastrichtian (Figure 7A).

### Latest Cretaceous dinosaur diversity and disparity in North America

Hadrosaurids are abundant in Late Cretaceous dinosaur assemblages of North America and are essential for understanding patterns of dinosaur diversity and extinction at the end of the Mesozoic. A number of studies suggest that the global pattern of dinosaur diversity is stable throughout the Late Cretaceous [1]–[3], while others argue for decreasing diversity during this interval [4]–[8]. A number of recent studies argue that alpha diversity of dinosaur faunas from the latest Maastrichtian of North America has been overestimated, and have emphasized the importance of ontogeny and variation for understanding the nature of morphological diversity in tyrannosaurids [9], [46], pachycephalosaurids [11], basal ornithopods [10], and ceratopsids [12]. When a similar perspective is applied to the hadrosaurid assemblage, the morphometric results presented here support the presence of only a single hadrosaurid species, *Edmontosaurus annectens*, in the latest Maastrichtian interval.

Our conclusions on edmontosaur systematics and biostratigraphy, together with recent revisions [9], [12], [46], have implications for the diversity dynamics of dinosaurs from the latest Campanian to the end of the Maastrichtian in North America. In the context of latest Cretaceous hadrosaurid diversity, our results suggest a drop in species richness during the well-sampled Maastrichtian interval, as both hadrosaurines and lambeosaurines co-occur in the Early Maastrichtian [18], [31], [47]. Recent revisions of ceratopsids [12] and the absence of centrosaurines in the latest Maastrichtian [48], suggests that the species-level diversity in these two dominant megaherbivore groups may have declined in the latest Cretaceous of North America. A similar pattern of decreasing species richness has also been suggested for small theropods [49], may also occur in small-bodied herbivores (e.g., pachycephalosaurs, [11]), and may well characterize North American dinosaur faunas in general [7], but further, more comprehensive research is needed to firmly establish the pattern of dinosaur diversity leading up the end Cretaceous extinction event.

Lower-level taxonomic assessments are important for interpreting diversity dynamics, however, they can often be subjective and controversial in nature, particularly with respect to dinosaurs (e.g., [12], [50]). Therefore, a quantitative disparity approach provides an alternative measure of morphological diversity that is independent of alpha taxonomy [51]. A preliminary disparity analysis of hadrosaurids from the latest Campanian to latest Maastrichtian time interval (73 to 65.5 Ma) based on a geometric morphometric dataset (Figure S3) reveals a notable decline in morphospace occupation and Foote's disparity from the early to late Maastrichtian that is directly linked to the absence of lambeosaurines (Figure 7B,C). Because chasmosaurines are the only remaining ceratopsids in the latest Maastrichtian and centrosaurines are absent [12], we predict a similar decline in the disparity of horned dinosaurs through the same time interval. Structural differences in the feeding apparatus between hadrosaurines and lambeosaurines [52], [53], as well as between chasmosaurines and centrosaurines [54], have been hypothesized to represent differences in feeding ecology between these major groups. Consequently, a probable Maastrichtian decline in hadrosaurid and ceratopsian species richness in North America coincides with a loss in morphological and ecological diversity in the megaherbivore faunal assemblage just prior to the end-Cretaceous extinction event.

## Supporting Information

Figure S1 **Vector plots of RW1 and RW2.** (A) Morphological changes along the first relative warp axis, from the positive to the negative spectrum. (B) Morphological changes along the second relative warp axis, from the negative to the positive spectrum.
(EPS)

Figure S2 **Box and whisker plots.** Groups are divided based on the temporal subsamples described in the text. In both plots, temporal subsamples are significantly different from each other (two tailed t-test: p≪0.01). The orange triangle with black outline represents the holotype of *A. copei* (AMNH 5730), and the orange triangle represents MOR 003.
(EPS)

Figure S3 **Materials, methods and results of the disparity analysis.** (A) Skulls of other hadrosaurids present between 73 and 65.5 Ma and included in the GM analysis. *Velafrons coahuilensis* is modified from Gates et al. [31]. Scale bar, 20 cm. (B) Landmarks used in the geometric morphometric analysis shown in figure 7B, and which form the basis to estimate morphological disparity. (C) Hadrosaurid disparity through the latest Cretaceous.
(TIF)

Table S1 **Linear measurements for all edmontosaur skulls examined in this study.**
(DOCX)

Table S2 **Loadings of the linear variables along the first three principal component axes.**
(DOCX)

Text S1 **Taxonomic history of edmontosaurs and institutional abbreviations.**
(DOC)
