# Supplementary figures and images for: Cranial Growth and Variation in Edmontosaurs (Dinosauria: Hadrosauridae): Implications for Latest Cretaceous Megaherbivore Diversity in North America
Source: PLoS One. 2011 Sep 28;6(9):e25186. doi: 10.1371/journal.pone.0025186 (PMC3182183; doi:10.1371/journal.pone.0025186)

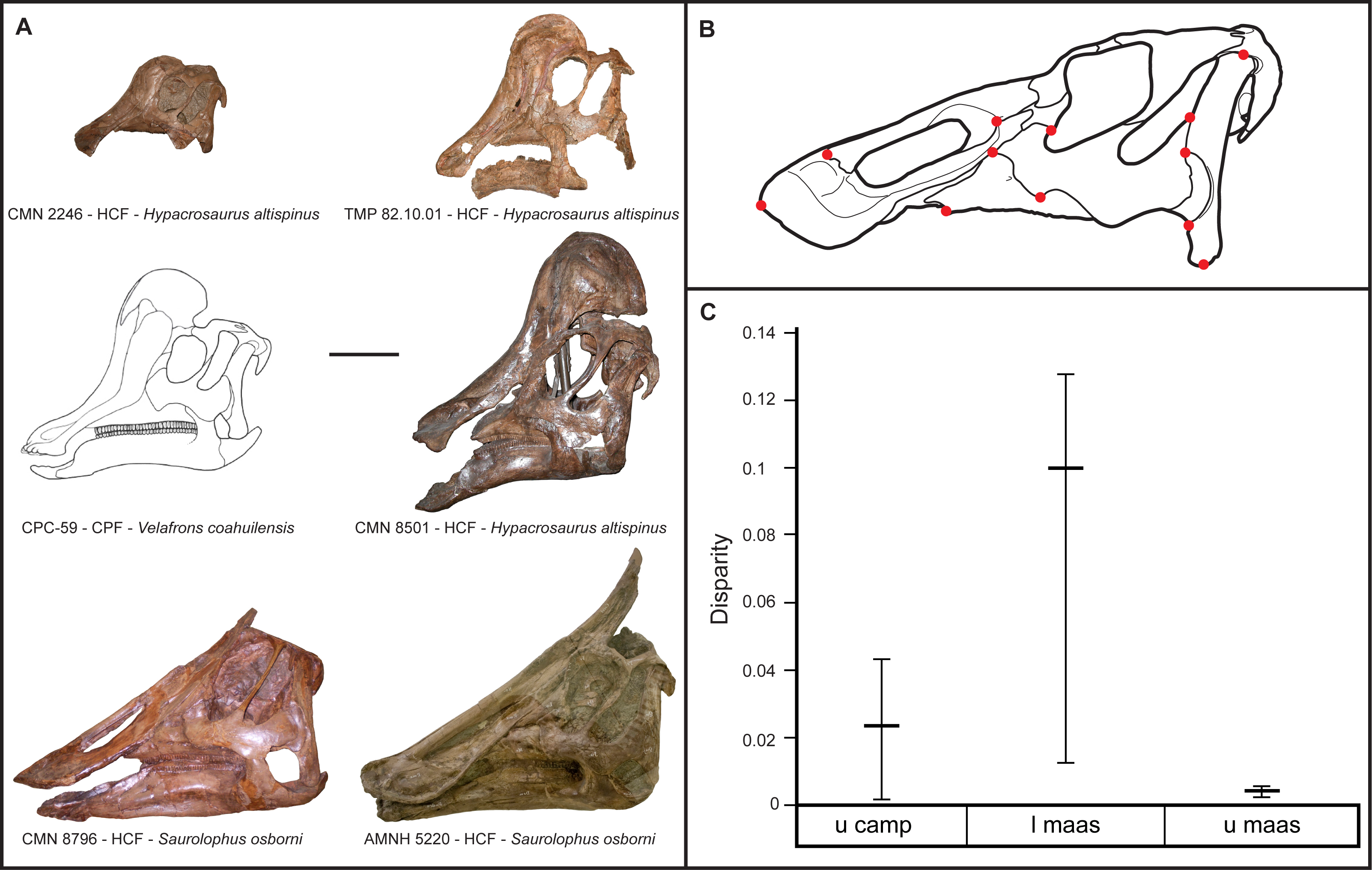

Supplement: Figure S3 — Materials, methods and results of the disparity analysis. (A) Skulls of other hadrosaurids present between 73 and 65.5 Ma and included in the GM analysis. Velafrons coahuilensis is modified from Gates et al. [31]. Scale bar, 20 cm. (B) Landmarks used in the geometric morphometric analysis shown in figure 7B, and which form the basis to estimate morphological disparity. (C) Hadrosaurid disparity through the latest Cretaceous. (TIF) [file pone.0025186.s003.tif]
