# Supplementary material for: Cranial Growth and Variation in Edmontosaurs (Dinosauria: Hadrosauridae): Implications for Latest Cretaceous Megaherbivore Diversity in North America
Source: PLoS One. 2011 Sep 28;6(9):e25186. doi: 10.1371/journal.pone.0025186 (PMC3182183; doi:10.1371/journal.pone.0025186)
Supplement: Table S1 — Linear measurements for all edmontosaur skulls examined in this study. (DOCX) [file pone.0025186.s004.docx]

Supplementary Table 1. Measurements taken from edmontosaur crania and used in PCA and bivariate allometric analyses. Measurement abbreviations: Den.L, dentary length; Dias.L, length of the edentulous region of the dentary; J.H, height of the jugal ventral phlange; J.L, jugal length; Max.H, maxilla height; Nar.L, naris length; Nar.V.L, narial vestibule length; Po.L, postorbital length; Prn.L, prenarial length; Q.H, quadrate height; Rm.W, width of the reflected margin of premaxilla; Sk.L, skull length, Sn.H, snout height.

|  | Previously Assigned to: | Sp. # | Age | 1. Sk.L | 2. Nar.L | 3. Prn.L | 4. Nar.V.L | 5. Rm.W | 6. Sn.H | 7. Max.H | 8. Q.H | 9. Po.L | 10. J.L | 11. J.H | 12. Den.L | 13. Dias.L | % MD |
| --- | --- | --- | --- | --- | --- | --- | --- | --- | --- | --- | --- | --- | --- | --- | --- | --- | --- |
| 1 | *Edmontosaurus regalis* | AMNH 5254 | C | - | - | - | - | - | - | 119 | 422 | 243 | 387 | 125 | 765 | 308 | 46.2 |
| 2 | *Edmontosaurus regalis* | NHM R8927 | C | 850 | 205 | 154 | 351 | 74 | 183 | 92 | 349 | 213 | 330 | 99 | 615 | 250 | 0 |
| 3 | *Edmontosaurus regalis* | CM 26259 | C | 810 | 220 | 141 | 358 | 71 | 181 | 84 | 353 | 191 | 294 | 95 | - | 225 | 7.7 |
| 4 | *Edmontosaurus regalis* | CMN 2288 | C | 1020 | 334 | 225 | 478 | 118 | 233 | 107 | 416 | 271 | 359 | 112 | 780 | 311 | 0 |
| 5 | *Edmontosaurus regalis* | CMN 2289 | C | - | - | - | - | - | 243 | 111 | 417 | 291 | 349 | 132 | 775 | 325 | 38.5 |
| 6 | *Edmontosaurus regalis* | FMNH 15004 | C | 1087* | 281 | 236 | 487 | 101 | 263 | 121 | 424 | 244 | 341 | 118 | 692 | 303 | 0 |
| 7 | *Edmontosaurus regalis* | ROM 801 | C | 1065 | 295 | 219 | 510 | 102 | 236 | 115 | - | - | 380 | 124 | 850 | - | 23.1 |
| 8 | *Edmontosaurus regalis* | USNM 12711 | C | 1040 | 257 | 252 | 463 | 106 | 217 | 99 | 422 | 270 | 339 | 136 | 752 | 327 | 0 |
| 9 | *Thespesius edmontoni* | CMN 8399 | C | 943 | 226 | 173 | 370 | 75 | 181 | 78 | 340 | 216 | 287 | 95 | 617 | 254 | 0 |
| 10 | *Edmontosaurus annectens* | BHI 2169 | M | 1040 | - | - | - | - | - | 87 | 389 | 225 | 358 | 97 | 775 | 340 | 38.5 |
| 11 | *Edmontosaurus annectens* | DMNH 1493^1^ | M | - | - | - | - | - | 215.8 | - | 395 | 217 | 350 | 95 | 745 | 335 | 46.2 |
| 12 | *Edmontosaurus annectens* | ROM 57100 | M | 1050 | 264 | 299 | 533 | 72 | 192 | 97.9 | 402 | 194 | 368 | 98 | 750 | 323 | 0 |
| 13 | *Edmontosaurus annectens* | SM R4050 | M | 960 | 274 | 263 | 471 | 74 | 170 | 95 | 388 | 187 | 315 | 121 | 676 | 330 | 0 |
| 14 | *Edmontosaurus annectens* | UCMP 128374^1^ | M | 1083 | - | 277 | 513 | 89 | 197 | 82 | 390 | 216 | 375 | 104 | 725 | 333 | 7.7 |
| 15 | *Edmontosaurus annectens* | UMMP 20000 | M | 1113 | 296 | 272 | 509 | 73* | 192 | 98 | 443 | 264 | 384 | 122 | 747 | 311 | 0 |
| 16 | *Edmontosaurus annectens* | USNM 3814 | M | 1078 | 301 | 285 | 569 | 92.5 | 190 | 107 | 417 | - | 393 | 108 | 785 | 358 | 7.7 |
| 17 | *Edmontosaurus annectens* | NCSM 23119* | M | 1037 | - | - | - | - | 196 | 94 | 370 | 181 | 367 | 121 | - | - | 46.2 |
| 18 | *Anatotitan copei* | AMNH 5730^2^ | M | 1175 | 263 | 316 | 567 | 60 | 163 | 98 | 328 | 220 | 345 | 107 | 841 | 353 | 0 |
| 19 | *Anatotitan copei* | CCR No# | M | 1193 | - | 373 | 573 | - | 208 | 101 | 410 | - | 345 | 97 | 788 | 388 | 30.8 |
| 20 | *Anatotitan copei* | MOR 003 | M | 1278 | 347 | 330 | 633 | 89 | 247 | 110 | - | 231 | 385 | 115 | - | - | 23.1 |
| 21 | *Edmontosaurus saskatchewanensis* | CMN 8509 | M | 781 | 197 | 159 | 348 | 56 | 151 | 67 | 330 | 197 | 285 | 73 | 575 | 234 | 0 |

- Missing data

* Measured using Image J

^1^ Measurements taken from a cast at the MOR

^2^ Measurements taken from a cast at the TMP

C Campanian

M Maastrichtian

MD Missing Data
