# Supplementary material for: Cranial Growth and Variation in Edmontosaurs (Dinosauria: Hadrosauridae): Implications for Latest Cretaceous Megaherbivore Diversity in North America
Source: PLoS One. 2011 Sep 28;6(9):e25186. doi: 10.1371/journal.pone.0025186 (PMC3182183; doi:10.1371/journal.pone.0025186)
Supplement: Table S2 — Loadings of the linear variables along the first three principal component axes. (DOCX) [file pone.0025186.s005.docx]

Supplementary Table 2. The loadings of the 13 measurements shown in figure 3A on the first three principal component axes, along with the percentage of variance explained by each axis.

|  | **Axis** | **PC1** | **PC2** | **PC3** |
| --- | --- | --- | --- | --- |
|  | **% Variation Explained** | **65.9** | **21.4** | **3.4** |
| 1 | Skull Length | -0.259 | 0.111 | -0.073 |
| 2 | Narial Length | -0.319 | -0.061 | -0.08 |
| 3 | Prenarial Length | -0.441 | 0.524 | 0.035 |
| 4 | Narial Vestibule Length | -0.354 | 0.231 | -0.043 |
| 5 | Reflected Premaxillary Margin Width | -0.291 | -0.561 | -0.079 |
| 6 | Snout Height | -0.239 | -0.347 | -0.211 |
| 7 | Maxilla Height | -0.274 | -0.21 | 0.239 |
| 8 | Quadrate Height | -0.171 | -0.112 | -0.062 |
| 9 | Postorbital Length | -0.149 | -0.244 | 0.532 |
| 10 | Jugal Length | -0.185 | 0.021 | -0.013 |
| 11 | Jugal Flange Height | -0.241 | -0.218 | 0.768 |
| 12 | Dentary Length | -0.239 | 0.074 | 0.028 |
| 13 | Diastema Length | -0.304 | 0.225 | -0.012 |
