# Supplementary material for: Cranial Growth and Variation in Edmontosaurs (Dinosauria: Hadrosauridae): Implications for Latest Cretaceous Megaherbivore Diversity in North America
Source: PLoS One. 2011 Sep 28;6(9):e25186. doi: 10.1371/journal.pone.0025186 (PMC3182183; doi:10.1371/journal.pone.0025186)
Supplement: Text S1 — Taxonomic history of edmontosaurs and institutional abbreviations. (DOC) [file pone.0025186.s006.doc]

**Supplement 1:**

**Taxonomic history of edmontosaurs**

As the first species diagnoses of hadrosaurians from the Edmonton and Lance Formations in the late 1800s and early 1900s were based on fragmentary, generally undiagnostic, material the following taxonomic history of edmontosaur species begins with the first synthesis of North American hadrosaurids by Lull and Wright [SS1].

Type specimens and provenance

**1. *Edmontosaurus regalis* Lambe 1917 [SS2]**

**Holotype.** CMN 2288

**Paratype.** CMN 2289

**Type Locality and Horizon.** CMN 2288, Red Deer River, Alberta, opposite the mouth of Three Hills Creek, 200 feet above the river level; Horseshoe Canyon Formation (=Edmonton Formation). CMN 2289, west side of the Red Deer River, 7 miles northwest of Morrin, 90 feet above the river level; Horseshoe Canyon Formation.

**2. *Anatosaurus annectens* (Marsh, 1892) [SS3]**

**Holotype.** USNM 2414

**Plesiotype.** YPM 2182

**Type Locality and Horizon.** USNM 2414, north of Lightning and east of Bull creeks; Lance Formation (high in the series). YPM 2182, south of Schneider and north of Greasewood creeks, near a smaller tributary of the Cheyenne River, Niobrara Co., Wyoming; Lance Formation (high in the series).

**3. *Anatosaurus copei* Lull and Wright 1942 [SS1]**

**Holotype.** AMNH 5730

**Plesiotype.** AMNH 5886

**Type Locality and Horizon.** AMNH 5730, Near the Moreau River, north of the Black Hills, South Dakota; Lance Formation. AMNH 5886, Crooked Creek, Montana; Lance Formation.

**4. *Anatosaurus saskatchewanensis* (Sternberg, 1926) [SS4]**

**Holotype.** CMN 8509

**Type Locality and Horizon.** Rocky Creek, Saskatchewan. Sec. 22, T. 1, R. 5, west of 3d principal meridian; Frenchman Formation (equivalent to the upper Lance/Hell Creek Formations).

**5. *Anatosaurus edmontoni* (Gilmore, 1924) [SS5]**

**Holotype.** CMN 8399

**Type Locality and Horizon.** On Michichi Creek, 5 miles from Drumheller, Red Deer River, Alberta; Horseshoe Canyon Formation.

**6. *Anatosaurus longiceps* (Marsh, 1890) [SS6]**

**Holotype.** YPM 616

**Type Locality and Horizon.** Niobrara County, Wyoming; Lance Formation.

Detailed historical synonymies

**1. Lull and Wright 1942**

*Edmontosaurus regalis* Lambe 1917 [SS2]

Referred Specimens.

| Sp. # | Locality | Formation | Age |
| --- | --- | --- | --- |
| CMN 2288 | Red Deer River, Alberta | Horseshoe Canyon | Latest Campanian |
| CMN 2289 | Red Deer River, Alberta | Horseshoe Canyon | Latest Campanian |
| ROM 801 (5167) | Red Deer River, Alberta | Horseshoe Canyon | Latest Campanian |
| USNM 12711 | Red Deer River, Alberta | Horseshoe Canyon | Latest Campanian |

*Anatosaurus annectens* (Marsh, 1892) [SS3]

*Claosaurus annectens* Marsh 1892 [SS3]

**Referred Specimens**.

| Sp. # | Locality | Formation | Age |
| --- | --- | --- | --- |
| USNM 2414 | Niobrara Co., Wyoming | Lance | Late Maastrichtian |
| YPM 2182 | Schneider, Wyoming | Lance | Late Maastrichtian |
| AMNH 5060 | Converse Co., Wyoming | Lance | Late Maastrichtian |

*Anatosaurus copei* Lull and Wright 1942 [SS1]

*Diclonius mirabilis* Cope 1883 [SS7]

Referred Specimens.

| Sp. # | Locality | Formation | Age |
| --- | --- | --- | --- |
| AMNH 5730 | Moreau River, S. Dakota | Lance | Late Maastrichtian |
| AMNH 5886 | Crooked Creek, Montana | Lance | Late Maastrichtian |

*Anatosaurus saskatchewanensis* (Sternberg, 1926) [SS4]

*Thespesius saskatchewanensis* Sternberg 1926 [SS4]

Referred Specimens.

| Sp. # | Locality | Formation | Age |
| --- | --- | --- | --- |
| CMN 8509 | Rocky Ck., Saskatchewan | Frenchman | Late Maastrichtian |

*Anatosaurus edmontoni* (Gilmore, 1924) [SS5]

*Thespesius edmontoni* Gilmore 1924 [SS5]

**Referred Specimens.**

| Sp. # | Locality | Formation | Age |
| --- | --- | --- | --- |
| CMN 8399 | Michichi Ck., Alberta | Horseshoe Canyon | Latest Campanian |
| ROM 867 (5851) | Red Deer River, Alberta | Horseshoe Canyon | Latest Campanian |

*Anatosaurus longiceps* (Marsh, 1890) [SS6]

*Trachodon longiceps* Marsh 1890 [SS6]

Referred Specimens.

| Sp. # | Locality | Formation | Age |
| --- | --- | --- | --- |
| YPM 616 | Niobrara Co., Wyoming | Lance | Late Maastrichtian |

**2. Brett-Surman 1979/Chapman and Brett-Surman 1990**

*Anatotitan copei* (Lull and Wright 1942) [SS1]

*Anatosaurus copei* Lull and Wright 1942 [SS1]

Referred Specimens.

| Sp. # | Locality | Formation | Age |
| --- | --- | --- | --- |
| AMNH 5730 | Moreau River, S. Dakota | Hell Creek | Late Maastrichtian |
| AMNH 5886 | Crooked Creek, Montana | Hell Creek | Late Maastrichtian |
| AMNH 5887 | Hell Creek, Montana | Hell Creek | Late Maastrichtian |
| CM 16520 |  |  |  |
| CCM No # | Montana | Hell Creek | Late Maastrichtian |

**Comments.** Diagnosis and list of referred specimens was provided in Chapman and Brett-Surman [SS8]. CM 16520 does not refer to any specimen at the Carnegie Museum. It is likely that the authors were referring to CM 1652, which is a partial postcranial specimen of a hadrosaurid, perhaps *Edmontosaurus annectens*, but the material is not diagnostic.

*Edmontosaurus regalis* and *Edmontosaurus edmontoni*

**Synonymy.** The authors do not specify which taxa are synonymous with either *E. regalis or E. edmontoni* but presumably these two species include all known *Anatosaurus* taxa described by Lull and Wright [S1], except for *Anatosaurus copei*.

**General Comments.** Brett-Surman [S9] provides no specific specimen numbers, but says:

“*'A'* coming off the *Edmontosaurus* lineage represents *Anatosaurus copei* only. This form will be redescribed as a new genus elsewhere. All other species of *Anatosaurus* are considered junior synonyms of *Edmontosaurs regalis* and *E.* *edmontoni.* There is a distinct possibility that these two species may be male and female respectively.” [S9; Fig. 1 caption, pg. 561].

**3. Weishampel and Horner 1990**

*Edmontosaurus regalis* Lambe, 1917 [S2]

*Trachodon atavus* Cope, 1871 [S10]

*Agathaumas milo* Cope, 1874 [S11]

**Locality Information.** Alberta, Canada: Horseshoe Canyon Formation; St. Mary River Formation; Scollard Formation; South Dakota, U.S.A.: Hell Creek Formation, Lance Formation; North Dakota, U.S.A.: Hell Creek Formation; Wyoming, U.S.A.: Lance Formation; Colorado, U.S.A.: Laramie Formation.

**Material.** Approximately 7 fully articulated skull and associated postcrania, 5-7 articulated skulls, associated skull elements.

**Age Range.** early-late Maastrichtian.

**Comments.** Although the authors refer to *Trachodon atavus*, we have been unable to find a single reference, which refers to its existence. The closest may be *Trachodon cavatus*, described by Cope [S10] from Greensand, New Jersey. *Trachodon cavatus* was considered a *nomen nudum* by Lull and Wright [S1].

*Edmontosaurus annectens* (Marsh, 1892) [S3]

*Claosaurus annectens* Marsh 1892 [S3]

*Thespesius* *edmontonensis* Gilmore, 1924 [S5]

**Locality Information.** Alberta, Canada: Scollard Formation; Saskatchewan, Canada: Frenchman Formation; Montana/South Dakota, U.S.A.: Hell Creek Formation; Wyoming, U.S.A.: Lance Formation; Colorado, U.S.A.: Laramie Formation.

**Material.** At least 5 articulated skull and associated postcranial skeletons, isolated skull material.

**Age Range.** Late Maastrichtian

**Comments.** Weishampel and Horner [S12] refer to *Thespesius edmontonensis*. This specific epithet is invalid, the original species description names *Thespesius edmontoni* [S5].

*Edmontosaurus saskatchewanensis* (Sternberg, 1926) [S4]

*Thespesius saskatchewanensis* Sternberg 1926 [S4]

**Locality Information.** Frenchman Formation, Saskatchewan, Canada

Late Maastrichtian, Complete skull, 3-4 partial skulls”

**Age Range.** Late Maastrichtian

*Anatotitan copei* (Lull and Wright, 1942) [S1]

*Anatosaurus copei* Lull and Wright 1942 [S1]

*Trachodon longiceps* Marsh 1890 [S6]

**Locality Information.** Hell Creek Formation, Montana, U.S.A.; Lance Formation, Hell Creek Formation, South Dakota, U.S.A.

**Material.** 2-3 articulated skulls, associated postcrania, isolated skull elements.

**Age Range.** Late Maastrichtian

**Comments.** Description of *Trachodon longiceps* was published in 1890, not 1897.

**4. Horner et al. 2004**

*Edmontosaurus regalis* Lambe, 1917 [S2]

*Trachodon atavus* Cope, 1871 [S10]

*Agathaumas milo* Cope, 1874 [S11]

**Locality Information.** Horseshoe Canyon Formation; St. Mary River Formation; Scollard Formation, Alberta, Canada; Hell Creek Formation, Montana, U.S.A.; Hell Creek Formation, Lance Formation, South Dakota, U.S.A.; Hell Creek Formation, North Dakota, U.S.A.; Lance Formation, Wyoming, U.S.A.; Laramie Formation, Colorado, U.S.A.

**Material.** Approximately 7 fully articulated skull and associated postcrania, 5-7 articulated skulls, associated skull elements.

**Age Range.** early-late Maastrichtian.

**Comments.** See previous Weishampel and Horner 1990 section for reference to *Trachodon atavus*. (

ope, 1874)

*Edmontosaurus annectens* (Marsh, 1892) [S3]

*Claosaurus annectens* Marsh 1892 [S3]

*Thespesius* *edmontonensis* Gilmore, 1924 [S5]

*Anatosaurus copei* Lull and Wright 1942 [S1]

*Trachodon longiceps* Marsh, 1890 [S6]

**Locality Information.** Scollard Formation, Alberta, Canada; Hell Creek Formation, Montana, U.S.A.; Hell Creek Formation, Lance Formation, South Dakota, U.S.A.; Lance Formation, Wyoming, U.S.A.; Laramie Formation, Colorado, U.S.A.

**Material.** At least 5 articulated skull and associated postcranial skeletons, isolated skull material.

**Age Range.** Late Maastrichtian

**Comments.** *Thespesius edmontonensis* = *Thespesius edmontoni*.

*Edmontosaurus saskatchewanensis* (Sternberg, 1926) [S4]

*Thespesius saskatchewanensis* Sternberg, 1926 [S4]

**Locality Information.** Frenchman Formation, Saskatchewan, Canada

**Material.** Complete skull, 3 or 4 partial skulls.

**Age Range.** Late Maastrichtian

**General Comments.** “As for *Edmontosaurus* itself, we regard it as the senior synonym of *Anatotitan* because we can find no unambiguous characters to separate these two taxa. Skull shape differences used by Brett-Surman (1979) to distinguish between these taxa are likely due to dorsoventral crushing of the *Anatotitan* skull.” [S13; pg. 460].

*Trachodon longiceps* Marsh 1890 from the Lance Formation of Wyoming was synonymized with *Anatotitan copei* in Weishampel and Horner [S12], and *Edmontosaurus annectens* in Horner et al. [S13]. The type material is based on a portion of the dentary toothrow, and although it is large and likely *Edmontosaurus*, the material is non-diagnostic and we therefore consider it a *nomen-dubium*.

**5. Prieto-Marquez 2010**

*Edmontosaurus regalis* Lambe, 1917 [S2]

**Locality Information.** Hell Creek Formation, Montana, North/South Dakota, U.S.A; Lance Formation, Wyoming, U.S.A.; Laramie Formation, Colorado, U.S.A.; Horseshoe Canyon Formation, St. Mary River Formation, Scollard Formation, Alberta, Canada.

**Age Range.** late Campanian – late Maastrichtian.

*Edmontosaurus annectens* (Marsh, 1892) [S3]

*Anatotitan copei* (Lull and Wright, 1942) [S1]

*Edmontosaurus saskatchewanensis* (Sternberg, 1926) [S4]

**Locality Information.** Hell Creek Formation, Montana, North/South Dakota, U.S.A.; Lance Formation, South Dakota, Wyoming, U.S.A.; Laramie Formation, Colorado, U.S.A.; Scollard Formation, Alberta, Canada; Frenchman Formation, Saskatchewan, Canada.

**Age Range.** Late Maastrichtian

**General Comments.** The author provides no specimen numbers, and all locality and age data is based on Horner et al. [S13].

“None of the characters used by Chapman & Brett-Surman (1990) to diagnose *Anatotitan copei* were unique or allowed distinction of this taxon from other hadrosaurids. Likewise, examination of the skull of AMNH 5730 revealed several areas with signs of postdepositional dorsoventral compression. For example, each dentary had a longitudinal ridge protruding laterally, probably produced by bending of the lateral surface of the bone (the same deformation ridge was present in the right, but not the left, dentary of the paratype specimen of *Edmontosaurus* *regalis* Lambe, 1917b, CMN 2289) (Fig. 1); the dorsal region of the left quadrate had a transverse fissure (Fig. 2A); and the left postorbital ‘inflation’ of its central body appeared crushed inwards (medioventrally) (Fig. 2B), with a nearly vertical indented line on the lateral bone surface. Aside from the very shallow skull, AMNH 5730 and 5886 were indistinguishable from any specimen of *Edmontosaurus* *annectens*. Therefore, *A. copei* was regarded as a junior synonym of *E. annectens* in the present study, in agreement with Horner *et al.* (2004).” [S14; pg. 444].

References

S1. Lull RS, Wright NF (1942) Hadrosaurian dinosaurs of North America. Geological Society of America, Special Papers 40.

S2. Lambe LM (1917) A new genus and species of crestless hadrosaur from the Edmonton Formation of Alberta. The Ottawa Naturalist XXXI: 65-73.

S3. Marsh OC (1892) Notice of new reptiles from the Laramie Formation. American Journal of Science, Series 3 XLIII: 449-453.

S4. Sternberg CM (1926) A new species of *Thespesius* from the Lance Formation of Saskatchewan. Canada Department of Mines Bulletin, geological series 44: 73-84.

S5. Gilmore CW (1924) A new species of hadrosaurian dinosaur from the Edmonton Formation (Cretaceous) of Alberta. Canada Department of Mines Bulletin, geological series 43: 13-26.

S6. Marsh OC (1890) Additional characters of the Ceratopsidae, with notice of new Cretaceous dinosaurs. American Journal of Science, Series 3 XXXIX: 418-426.

S7. Cope ED (1883) On the characters of the skull in the Hadrosauridae. Proceedings of the Academy of Natural Sciences of Philadelphia XXXV: 97-107.

S8. Chapman RE, Brett-Surman MK (1990) Morphometric observations on hadrosaurid ornithopods. In: Carpenter K, Currie PJ, editors. Dinosaur systematics: perspectives and approaches: Cambridge University Press. pp. 163-177.

S9. Brett-Surman MK (1979) Phylogeny and palaeobiogeography of hadrosaurian dinosaurs. Nature 277.

S10. Cope ED (1871) Supplement to the "Synopsis of the extinct Batrachia and Reptilia of North America". Proceedings of the American Philisophical Society 12: 41-52.

S11. Cope ED (1874) Report on the stratigraphy and Pliocene vertebrate palaeontology of norther Colorado. United States Geological and Geographical Survey Terrain Bulletin 1: 9-28.

S12. Weishampel DB, Horner JR (1990) Hadrosauridae. In: Weishampel DB, Dodson P, Osmolska H, editors. The dinosauria. Berkeley, California: University of California Press. pp. 534-561.

S13. Horner JR, Weishampel DB, Forster CA (2004) Hadrosauridae. In: Weishampel DB, Dodson P, Osmolska H, editors. The dinosauria (2nd ed). 2nd Ed. ed. Berkeley, CA: University of California Press. pp. 438-463.

S14. Prieto-Márquez A (2010) Global phylogeny of hadrosauridae (Dinosauria: Ornithopoda) using parsimony and Bayesian methods. Zoological Journal of the Linnean Society 159: 435-502.

**Institutional Abbreviations**

AMNH American Museum of Natural History, New York, U.S.A.

BHI Black Hills Institute of Geological Research, Hill City, South Dakota, U.S.A.

CCM Carter County Museum, Ekalaka, Montana, U.S.A.

CM Carnegie Museum of Natural History, Pittsburgh, Pennsylvania, U.S.A.

CMN Canadian Museum of Nature, Ottawa, Ontario, Canada

DMNH Denver Museum of Nature and Science, Denver, Colorado, U.S.A.

FMNH Field Museum of Natural History, Chicago, Illinois, U.S.A.

LACM Natural History Museum of Los Angeles, Los Angeles, California, U.S.A.

MOR Museum of the Rockies, Bozeman, Montana, U.S.A.

NCSM North Carolina Museum of Natural Sciences, Raleigh, North Carolina, U.S.A.

NHM Natural History Museum, London, U.K.

ROM Royal Ontario Museum, Toronto, Ontario, Canada

SM Senckenberg Museum, Frankfurt, Germany

TMP Royal Tyrrell Museum of Palaeontology, Drumheller, Alberta, Canada

UCM University of Colorado Museum, Boulder, Colorado, U.S.A.

UCMP University of California Museum of Paleontology, Berkeley, California, U.S.A.

UMMP University of Michigan Museum of Paleontology, Ann Arbor, Michigan, U.S.A.

USNM National Museum of Natural History, Smithsonian Institute, Washington D.C., U.S.A.

YPM Yale Peabody Museum of Natural History, New Haven, Connecticut, U.S.A.
